# Antiepileptic and Antidepressive Polypharmacy in Patients with Multiple Sclerosis

**DOI:** 10.1155/2015/317859

**Published:** 2015-06-29

**Authors:** Georg Anton Giæver Beiske, Trygve Holmøy, Antonie Giæver Beiske, Svein I. Johannessen, Cecilie Johannessen Landmark

**Affiliations:** ^1^Department of Life Sciences and Health, Programme for Pharmacy, Oslo and Akershus University College, 0167 Oslo, Norway; ^2^Department of Neurology, Akershus University Hospital, 1478 Lørenskog, Norway; ^3^Institute of Clinical Medicine, University of Oslo, 0316 Oslo, Norway; ^4^MS Rehabilitation Centre, 1487 Hakadal, Norway; ^5^The National Center for Epilepsy, Oslo University Hospital, Sandvika, 0424 Oslo, Norway; ^6^Department of Pharmacology, Oslo University Hospital, 0424 Oslo, Norway

## Abstract

*Objective*. Patients with multiple sclerosis (MS) are often suffering from neuropathic pain. Antiepileptic drugs (AEDs) and tricyclic antidepressants (TCAs) are commonly used and are susceptible to be involved in drug interactions. The aim of this retrospective study was to investigate the prevalence of use of antiepileptic and antidepressive drugs in MS patients and to discuss the theoretical potential for interactions. *Methods*. Review of the medical records from all patients treated at a dedicated MS rehabilitation centre in Norway between 2009 and 2012. *Results*. In total 1090 patients attended a rehabilitation stay during the study period. Of these, 342 (31%; 249 females) with mean age of 53 (±10) years and EDSS 4.8 (±1.7) used at least one AED (gabapentin 12.7%, pregabalin 7.7%, clonazepam 7.8%, and carbamazepine 2.6%) or amitriptyline (9.7%). Polypharmacy was widespread (mean 5.4 drugs) with 60% using additional CNS-active drugs with a propensity to be involved in interactions. Age, gender, and EDSS scores did not differ significantly between those using and not using AED/amitriptyline. *Conclusion*. One-third of MS patients attending a rehabilitation stay receive AED/amitriptyline treatment. The high prevalence of polypharmacy and use of CNS-active drugs calls for awareness of especially pharmacodynamic interactions and possible excessive adverse effects.

## 1. Introduction

Neuropathic pain affects the quality of life and a recent meta-analysis of pain in MS reported a pooled prevalence of 63%, with estimates ranging from 29% to 86% [1, 2]. Treatment of neuropathic pain is associated with adverse reactions, especially long-term treatment where tolerance and dependence issues are concerned. Antiepileptic drugs (AEDs) such as carbamazepine, gabapentin, and pregabalin and the tricyclic antidepressant (TCA) amitriptyline are recommended and frequently used in the management of neuropathic pain [3–5]. AEDs are among the most susceptible drugs to be involved in pharmacokinetic as well as pharmacodynamic interactions [6]. Patients with MS often use several types of CNS-active drugs; yet little research has been done to highlight potential polypharmacy issues. There are no prevalence studies that estimate the extent of use of AEDs in patients with MS.

In Norway, the MS prevalence is about 203 per 100,000 [7]. Epilepsy has a prevalence of 0.7–1% worldwide [8]. There is an increased risk of developing epilepsy related to other neurological diseases as MS, and a prevalence of epilepsy and MS of 2.2% has been estimated [9]. During a 40-year follow-up of patients with MS in Norway, the risk of having active epilepsy was increased fourfold [10]. The use of AEDs to treat epilepsy in patients with MS is therefore also a treatment concern. Depression is one of the most common mental disorders, and, in the US, the prevalence is estimated to be 8.3% of lived years with disability [11]. Recently, it was shown that 10.7% of patients with MS (*n* = 75) were taking antidepressants [12].

The aim of this retrospective study was to investigate the prevalence of use of antiepileptic and antidepressive drugs in MS patients at a referral center and furthermore to discuss the theoretical potential for interactions, which may have a large impact on the pharmacological treatment of patients with MS.

## 2. Material and Methods

### 2.1. Study Population

This retrospective prevalence study was performed at the MS Rehabilitation Centre in Hakadal, Norway, to investigate the pharmacological treatment of patients admitted to the centre, based on data from the medical records. The centre is part of the Norwegian specialist healthcare service and patients are referred by their doctor to the centre for a four-week rehabilitation stay. Although any patient may be referred to the centre, more patients living in the southern, eastern, and northern health regions are referred as these regions have written agreements with the centre. The main intake criteria at the centre are the need for specialized interdisciplinary rehabilitation and potential for improvement during the stay. All patients are examined by a liaison neurologist from Akershus University Hospital at the beginning and end of their stay. All medical records from examinations of patients at the end of their stay in the period 01.01.2009–31.12.2012 were reviewed. Information on patients treated with at least one AED or amitriptyline was collected to investigate the suspected polypharmacy issues and potential for pharmacokinetic and pharmacodynamic interactions.

### 2.2. Inclusion Criteria

The study inclusion criteria were MS-diagnosis and use of at least one AED or amitriptyline, the only antidepressant with an indication for neuropathic pain in Norway. AEDs were defined as any drug with ATC code N03A (Anatomical Therapeutic Chemical classification) [16]. Some patients had more than one stay at the centre during the inclusion period. In those cases the most recent stay was chosen and previous stays were disregarded.

### 2.3. Data Handling and Analyses

Data for the patient group as a whole were considered, no interventions were performed, and follow-up data for the individual patients were not available. For every included patient, the following was registered: age, gender, Expanded Disability Status Scale (EDSS) score, and all current medications including dosages. Comorbid diagnoses such as bipolar disorder, depression, epilepsy, and migraine were also registered among patients using an AED or amitriptyline. To investigate and discuss the theoretical potential for drug interactions, AEDs and amitriptyline were categorized as Group I drugs, and other concomitantly used CNS-active drugs were categorized as Group II drugs. For Group I drugs the indication was collected. Group II drugs were considered especially relevant for potential interactions (mostly pharmacodynamic) with AEDs and amitriptyline. To study the use of those drugs in more detail, they were divided based on their mechanism of action: *α*_2_-blockers, GABA_B_ agonists, benzodiazepines, opioids, selective serotonin reuptake inhibitors (SSRI), and selective noradrenaline reuptake inhibitors (SNRI). Potential groups containing less than five patients were disregarded. This was done with the intention to increase awareness of the possible clinical consequences in practice and will be discussed.

If the medical record included an EDSS score, the EDSS score together with the patient's gender and age was collected, even if the patient failed to meet the inclusion criteria. This was done to provide a means to characterize the total patient population with an EDSS profile.

All patient data was stored anonymously in the database. The study was approved by the local ethics committee.

Statistical analyses included Fischer's *t*-test for binomial distributions, Mann-Whitney test for comparing nonparametric data (EDSS scores), and Student's *t*-test when comparing normally distributed data. A significance level of *p* < 0.05 was chosen.

## 3. Results

### 3.1. Study Population

A total of 1090 MS patients underwent a rehabilitation stay during the study period.  Table 1 shows patients' characteristics in detail.

Twenty (1.8%) of the patients had a diagnosis of epilepsy. Less than five of the patients using amitriptyline or any other tricyclic antidepressant had a stated diagnosis of depression in their medical records.

### 3.2. Use of Antiepileptic Drugs and Antidepressants

The use of AEDs, amitriptyline, and other CNS-active drugs is shown in Table 2. In total 342 (31%; 249 females) patients with mean age of 53 (±10) years and EDSS 4.8 (±1.7; *n* = 196) used at least one AED (295; 27%) or amitriptyline (106; 10%). There were 89 (26%) patients who used more than one AED or amitriptyline. Age, gender, and EDSS scores did not differ significantly between those using and not using AED/amitriptyline.

Dosage variability was extensive (Table 2) and varied between 18-fold for gabapentin, 12-fold for pregabalin and clonazepam, and 4.5-fold for carbamazepine.

In addition to the above-mentioned AEDs primarily used in neuropathic pain, seven other AEDs were used to treat epilepsy and other conditions (mood disorders including bipolar disorder, migraine, and pain), as shown in Table 2. No TCA other than amitriptyline was used for treatment of pain.

Twenty-nine percent of the patients using AEDs and/or amitriptyline were 60 years and above (*N* = 100). The average gabapentin dosage for these patients was 19% lower than for younger patients, 1277 (±687) and 1578 (±799) mg, respectively (*p* < 0.05). A similar tendency was seen with pregabalin, although nonsignificant.

### 3.3. Polypharmacy Aspects and Drug Interactions

**Figure 1:**
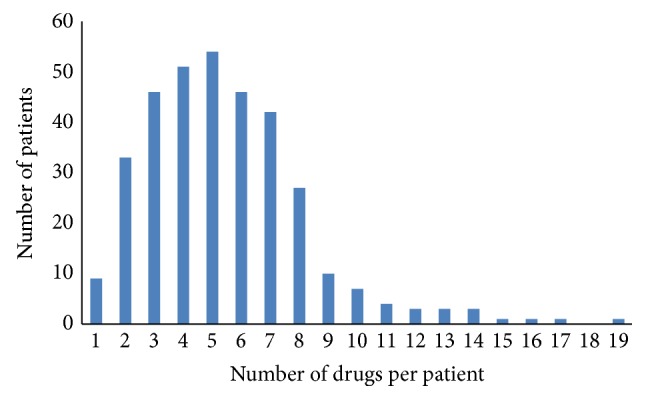
Drug count distribution among patients receiving antiepileptic and/or tricyclic antidepressant drugs (*N* = 342).

On average, patients receiving AED and/or amitriptyline treatment were using a total of 5.4 (range 1–19) different prescription drugs; 203 (59%) used 5 or more drugs and 24 (7%) used 10 drugs or more (Figure 1).

The AEDs and amitriptyline (Group I) were mostly used to treat neuropathic pain. The potential of drug interactions for other concomitantly used CNS-active drugs (Group II) was reviewed. Among the patients using one or more drugs in Group I, 204 (60%) also used one or more drugs in Group II. Drugs often used to treat mood disorders (*α*_2_-blockers, SSRI and SNRI) were used by 71 (21%) of the patients using drugs in Group I.

Thirty-eight percent of the patients using AEDs/amitriptyline (*N* = 130) were also receiving disease-modifying treatment (Table 1).

**Figure 2:**
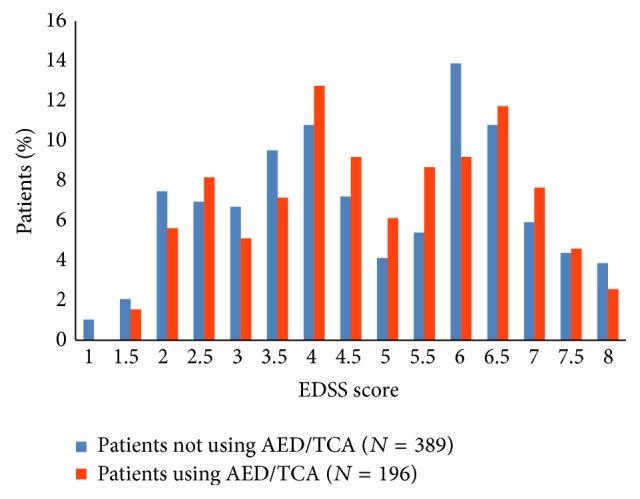
Expanded Disability Status Scale score distribution of the MS Rehabilitation Centre population (*N* = 585) divided into two groups based on the use of antiepileptic drugs (AED) and/or amitriptyline (TCA).

Figure 2 shows the wide distribution of functional levels in the MS Rehabilitation Centre population divided into two groups based on the use of AED/amitriptyline.

## 4. Discussion

In this study we demonstrate a wide use of AEDs and amitriptyline in a large population of patients with MS. Furthermore, the extent of polypharmacy in this patient population was high and 60% used additional CNS-active drugs. This forms the basis to discuss and evaluate the theoretical possibilities for interactions that may have a large impact on the treatment of the individual patient and calls for careful monitoring.

### 4.1. Study Population

The results in the present study reflect clinical use of AEDs and amitriptyline to treat pain in MS in a large population comprising about 10% of the patients with MS in Norway [7]. The female-to-male ratio and average age of the study population were comparable to the whole Norwegian MS population (2.4 versus 2.2 and 53 versus 51 years, resp.) [7]. The number of patients with an epilepsy diagnosis in this MS population of 1.8% was closer to the general population than previously reported [9].

### 4.2. Use of Antiepileptic Drugs and Amitriptyline

The present results show that the use of AEDs and amitriptyline was extensive in patients across all functional levels. The wide use of gabapentin, pregabalin, and amitriptyline for the treatment of pain in patients with MS is in line with international recommendations [3, 4]. Gabapentin and pregabalin have equal pharmacodynamic actions and are predominantly utilized in treatment of neuropathic pain, rather than epilepsy in Norway [5, 13]. We have recently shown that the pharmacokinetic variability of both pregabalin and gabapentin in clinical practice is more than 100-fold [17]. Head-to-head comparisons of the two are lacking, and even though pregabalin is considerably more expensive, the cost-efficiency is similar [18]. Clonazepam is often used to treat spasms and may be given in a small dose in addition to other drugs to treat pain. The enzyme inducer carbamazepine is still the AED of choice in trigeminus neuralgia, although skin rash is a common side effect and major reason for discontinuation [19]. Carbamazepine was frequently used in patients with MS and epilepsy, which calls for attention in combination with other drugs metabolized in the liver. Other AEDs are used only rarely in the treatment of pain [5].

There were large differences in dosages described for pregabalin, gabapentin, and clonazepam. For gabapentin, the pharmacokinetic absorption process is variable between patients and intraindividually due to limited absorption capacity [20], which explains why some patients will need a higher dose to reach a therapeutic serum concentration. Age also contributes to pharmacokinetic variability. Patients 60 years and above generally have lower renal clearance and may therefore require lower dosages of drugs cleared renally, such as gabapentin and pregabalin, to avoid adverse reactions. Age had probably been taken into account in these patients, since the dosages of both drugs were approximately 20% lower in patients 60 years and above than in younger patients.

Altogether 11 different AEDs were used by patients with MS, and in many cases this reflects comorbidity and possibly also off-label use of lamotrigine, valproic acid, and oxcarbazepine, since these drugs do not have a license indication for pain in Norway. These drugs have shown clinical efficacy in neuropathic pain and are approved for this indication in other countries [13].

### 4.3. Polypharmacy Aspects

We have shown that polypharmacy was widespread in this patient population. Although AEDs are well known to be involved in pharmacokinetic interactions, this is not of particular concern for MS patients since they mainly used newer AEDs (pregabalin and gabapentin) with less propensity to interact than the older enzyme inducers and inhibitors [6, 14]. Pharmacodynamic interactions are of greater concern since half of the patients used an opioid, a benzodiazepine or baclofen (GABA_B_ agonist) in addition to their AED/amitriptyline therapy.

Patients using an AED and/or amitriptyline used 5.4 drugs on average, which is regarded as a considerable drug load, and altogether up to 19 concomitant drugs were listed in one patient. The association between polypharmacy (≥5 drugs) and adverse drug reactions is well known and calls for attention and close monitoring [21]. The clinical consequence of a specific drug interaction may be difficult to predict in the individual patient. Different aspects of the pharmacological treatment may be affected, such as the drug efficacy or the adverse reaction profile. All drugs listed among the Group I and Group II drugs can cause sedation and general CNS depression. Considering that fatigue is one of the most common MS symptoms affecting approximately 75% of patients [22, 23], these pharmacodynamic interactions are likely to be clinically relevant.

### 4.4. Clinical Considerations

In a study of 142 MS patients in Norway with a pain prevalence of 65%, only one-third were receiving treatment [24]. Our finding that one-third of the MS patients at the MS Rehabilitation Centre receive AED/amitriptyline treatment, across all functional levels and ages, adds to this.

Rational combination of several CNS-active drugs with extensive pharmacological variability requires careful consideration of clinical as well as basic pharmacological aspects of pharmacotherapy. Treatment strategies in MS are changing, and the clinical experience regarding efficacy and tolerability of newly introduced drugs is limited [25]. A reduction in total drug load will probably result in less adverse reactions caused by pharmacodynamic interactions in polypharmacy patients, possibly improving quality of life [26].

The dosages should be reduced when several CNS-active drugs are given concomitantly to avoid excessive adverse reactions such as sedation, dizziness, and cognitive impairment. This is of special importance in patients 60 years and above due to extensive pharmacokinetic variability and increased sensitivity to CNS-active drugs. For example, pregabalin combined with zopiclone or opioids increases the risk of pharmacodynamic interaction and sedation, as well as other CNS-depressant effects, which have been reported in WHO's VigiBase in relation to abuse, tolerance, and dependence [27, 28].

The implementation of therapeutic drug monitoring (TDM) may contribute to an optimization of drug therapy in the individual patient by serum concentration measurements. TDM is used as a tool for tailoring the treatment with AEDs in epilepsy, as well as other indications, due to extensive pharmacokinetic variability between patients, challenges to handle drug interactions, and problems with adherence and for quality assurance of the treatment [29, 30].

The MS Rehabilitation Centre in Hakadal is a rehabilitation centre and, therefore, the most disabled and the oldest patients with MS are not included in the study population. To assess the selection bias we compared the study population's demographic data and EDSS scores with regional MS populations. We found that the study population has a similar functional level as the general MS population in one Norwegian county [31]. Peaks at scores 4.0 and 6.5 as seen in Figure 2 are typical for cross-sectional studies of MS populations, reflecting slower disease progression at these functional levels [31].

### 4.5. Methodological Considerations

Limitations of the study include the fact that the medical records may be incomplete. Thus, neither disease duration nor diagnosis of neuropathic pain was systematically reviewed, and a prevalence of neuropathic pain or its relationship with disease duration could not be estimated. Neuropathic pain was assumed as indication for the use of an AED/amitriptyline when no other indication was mentioned, based on the given indications in the summary of product characteristics and on the extensive use of gabapentin and pregabalin in neuropathic pain in Norway [5]. The records were, however, suitable to gain information on ongoing pharmacological treatment. The dosages recorded were assumed to be at steady state conditions, although some patients could have been at dosage titration. Furthermore, the study was retrospective, and efficacy and tolerability were not considered. The data were collected before cannabinoids were introduced for treatment of MS symptoms in Norway.

## 5. Conclusions

In this study we demonstrate a wide use of AEDs and amitriptyline in a large population of patients with MS (31%). Polypharmacy was widespread with 60% using additional CNS-active drugs with a propensity to be involved in interactions (5.4 drugs on average). One-third of the patients were 60 years and above, which is a patient group that needs especially careful pharmacological considerations. These results show that the high prevalence of use of CNS-active drugs calls for awareness of especially pharmacodynamic interactions and possible excessive adverse effects in patients with MS.

## Figures and Tables

**Table 1:** Patients' characteristics.

Study population	Description
Number of patients (*N* = 1090)	AED/amitriptyline: 342 (31%; 249 females)
Gender distribution	Mean age 53 (±10) years
Age distribution	Patients 60 years and above (29%; *N* = 100)
Range	21–73 years

EDSS score	MS Rehabilitation Centre population: mean 4.8 (±1.8, *N* = 585) AED/amitriptyline: mean 4.8 (±1.7; *N* = 196)

Polypharmacy	AED/amitriptyline: 5.4 (range 1–19) different prescription drugs 203 (59%) used 5 or more drugs 24 (7%) used 10 drugs or more

Comorbid disorders, epilepsy	MS Rehabilitation Centre population: *N* = 20 (1.8%), diagnosis of epilepsy
Mood disorder	AED/amitriptyline: *N* = 71 (21% used either *α*_2_-blocker, SNRI or SSRI)

Use of disease-modifying drugs	AED/amitriptyline: *N* = 130 (38%) Natalizumab: *N* = 40 (12%) Glatiramer acetate: *N* = 37 (11%) Interferon-beta: *N* = 36 (11%) Mitoxantrone: *N* = 7 (2%) Fingolimod: *N* = 6 (2%) Ocrelizumab: *N* = 1 (0.3%)

**Table 2:** AEDs and amitriptyline in pain and other indications 2009–2012 (*N* = 342) and potential drug interactions.

Drug	*N* (%)	Average dosage (mg)	Range (mg)	Indication	Route of elimination	Propensity to interact	Possible pharmacodynamic interactions
Group I: antiepileptic drugs (AEDs) (acting by inhibiting voltage gated sodium or calcium channels or as GABAergic drugs) or amitriptyline (TCA) (inhibiting reuptake of serotonin and noradrenaline^*∗∗*^)							
Gabapentin (AED)	138 (40.3)	1491	300–3600	Epilepsy (2) Pain or spasms^*∗*^ (136)	Renal	Very low	*Excessive CNS-sedation involving sedation, dizziness, fatigue, cognitive impairment, and so forth within GroupI or in combination with GroupII *
Clonazepam (AED)	85 (24.9)	1	0.25–3	Epilepsy (1) Pain or spasms^*∗*^ (84)	Hepatic CYP3A4	Moderate Metabolism inducible	
Pregabalin (AED)	24.6 (7.7)	448	50–900	Epilepsy (1) Pain or spasms^*∗*^ (83)	Renal	Very low	
Carbamazepine (AED)	28 (8.2)	469	200–900	Epilepsy (6) Bipolar disorder (1) Pain or spasms^*∗*^ (21)	Hepatic CYP3A4	Substantial Induces CYP3A4/2C9/1A2	
Lamotrigine (AED)	14 (4.1)	157	75–300	Epilepsy (6) Bipolar disorder (3) Pain or spasms^*∗*^ (5)	Hepatic UGT1A4 UGT2B7	Substantial	
Valproate (AED)	8 (2.3)	1012	600–1500	Epilepsy (5) Bipolar disorder (1) Pain or spasms^*∗*^ (1)	Hepatic CYP2C9/19/ 2A6/B6 oxidases	Substantial	
Levetiracetam (AED)	5 (1.5)	1200	500–2000	Epilepsy (3) Pain or spasms^*∗*^ (2)	Esterases in blood	Very low	
Oxcarbazepine (AED)	3 (0.9)	1080	600–1440	Epilepsy (1) Pain or spasms^*∗*^ (2)	Hepatic Arylketone reductase	Moderate	
Phenytoin (AED)	2 (0.6)	150	100–200	Epilepsy (2)	Hepatic CYP2C9/ 2C19	Substantial	
Topiramate (AED)	1 (0.3)	100	NA	Epilepsy (1)	Hepatic CYP isoenzymes	Substantial	
Phenobarbital (AED)	1 (0.3)	45	NA	Epilepsy (1)	Hepatic CYP2C9/ 2C19/E1	Moderate	
Amitriptyline (TCA)	106 (31.0)	29	5–75	Pain or spasms^*∗*^ (106)	Hepatic CYP2D6 2C19 3A4	Moderate Metabolism may be induced/ inhibited	

Group II: Pharmacodynamic interactions							
GABA_B_ agonist Opioids Benzodiazepines (GABA_A_ agonist) SSRI/SNRI *α*_2_-blockers	85 (24.9) 76 (22.2) 66 (19.9) 64 (18.7) 11 (3.2)	60% used at least one Group II drug in combination with a Group I drug, giving rise to a potential for drug interactions, where pharmacodynamic interactions are of most clinical relevance based on the data presented above. All drug classes result in a reduction in CNS-excitation based on their mechanisms of actions. Pharmacokinetic interactions are of limited importance quantitatively, since the AEDs most commonly used here have a low propensity to interact with pharmacokinetic processes					*Excessive CNS-sedation involving sedation, dizziness, fatigue, cognitive impairment, and so forth within GroupII or in combination with GroupI *

^*∗*^Pain/spasms were reported in the medical records or assumed when no other indications or comorbidities were reported.

^*∗∗*^Antagonism at other receptors causing adverse effects; histaminergic, noradrenergic, and muscarinergic receptors.

The data are based on [6, 13–15]. TCA: tricyclic antidepressant; AED: antiepileptic drug; VGCC: voltage gated calcium channels; VGSC: voltage gated sodium channels.
